# Somatic and psychiatric aspects of complications outside the surgical area in orthognathic surgery: A retrospective study of 429 patients

**DOI:** 10.4317/medoral.27718

**Published:** 2025-11-22

**Authors:** Sakari Kettunen, Evelina Metsäranta, Olli-Pekka Lappalainen, Jussi Furuholm, Johanna Snäll

**Affiliations:** 1 Department of Oral and Maxillofacial Diseases, University of Helsinki and Helsinki University Hospital, Helsinki, Finland

## Abstract

**Background:**

We explored postoperative non-surgical site complications in orthognathic surgery (OS) and investigated associations between outcome and patient- and surgery-related variables.

**Material and Methods:**

This single-centre (Department of Oral and Maxillofacial Diseases, Helsinki University Hospital, Helsinki, Finland) retrospective study included patients 18 years undergoing OS between January 2016 and December 2022. Data were manually reviewed from the hospital database. Associations with the outcome were analysed using SPSS software (IBM Corporation, 28.0.0.0).

**Results:**

Of 429 patients, 16 (3.7%) had a non-surgical site complication, and a total of 19 complications were recognized. A potentially life-threatening somatic complication occurred in 0.7% of patients. The most common complication was severe psychiatric morbidity, constituting 37% of all recognized complications. In the univariate and multivariable regression model, preoperative long-term disease (aOR 4.820; 95% CI 1.541-15.071, p=.007) and alcohol/substance abuse (p=.031) predicted the outcome independently. No other evaluated variables were associated with the outcome.

**Conclusions:**

The results suggest that severe general complications are rare and are associated with patients' long-term diseases. Severe psychiatric complications comprised a significant proportion of all recognized complications. Attention should be paid to patients' somatic and psychiatric status at all stages of treatment.

## Introduction

Orthognathic surgery (OS) can be used to treat patients whose dentofacial deformities are too severe for orthodontic camouflage or those who are too old for growth adjustment (1). Improved breathing (2), eating (3 , 4), and swallowing (4) are expected outcomes of OS, addressing the functional deficiencies brought on by skeletal misalignment of the jaws. Improvements in both oral health-related quality of life (5) and psychological and social quality of life (6) have been reported after OS. Nevertheless, since OS is a major maxillofacial procedure, complications within and outside the surgical site are possible.

Common surgical site complications related to OS have been widely described in the literature. These complications include surgical site infections or delay in surgical site healing (7), skeletal relapse or malocclusion (7 , 8), neurosensory deficits (7 - 10), and temporomandibular joint disorders (TMDs) (7 , 10 , 11). The risk and extent of complications have been described to be enhanced in patients with anatomical irregularities (12 , 13), and smoking has been reported to be a significant risk factor for infections post-operatively (14). Systemic inflammatory response syndrome (SIRS) has also been reported as a risk factor for OS-related complications. (15)

Complications in OS also occur outside the surgical site. These complications are rare but can be life-threatening. Extensive bleeding (.002-1.1%) (12 , 16 - 19), pneumonia (.5%) (20 , 21), lung atelectasis (.1%) (18), pneumothorax (.038%) (22 , 23), deep venous thrombosis (.05-.1%)(18 , 24), and pulmonary embolism (.02-.85%) (18 , 24 - 26) have been described in OS patients. Psychiatric morbidity or worsening of a pre-existing condition following OS has been reported as well (20). Single cases of very rare complications, such as cerebral hypoxia, injury to certain cranial nerves, and blindness, have been described (27 , 28). Death is seldom reported, however, Van de Perre et al. (18) noted one case (.05%) in their study of 2049 patients.

Numerous surgical site complications as well as issues outside the operative area have been identified based on prior research. We postulated that there may be other general complications of which we are still unaware. For successful treatment, a thorough understanding of the various complications outside the surgical site and factors influencing these is required. To the best of our knowledge, severe psychiatric complications are underreported, and further research is needed regarding psychiatric morbidity in OS. This study aimed to investigate complications outside the surgical site. We hypothesized that we would identify new complications warranting medical care or intervention.

## Material and Methods

Study design

A single-centre retrospective cohort study was conducted at the Department of Oral and Maxillofacial Diseases, Helsinki University Hospital, Finland, and data on patients undergoing OS between 1 January 2016 and 31 December 2022 were reviewed. Data were retrieved from electronic patient records based on surgical procedure codes.

Inclusion and exclusion criteria

Patients aged at least 18 years who received bilateral sagittal split (BSSO), Le Fort I, or bimaxillary-osteotomy and who had 6 months of postoperative follow-up were included in the study. Patients with oral cancer, developmental disability, intellectual disabilities, or secondary surgery for previous fracture or other maxillofacial surgery were excluded.

Study variables

The outcome variable was a general surgical complication defined as non-surgical site complication requiring medical treatment or intervention. The complications included postoperative complications within 90 days of the initial surgery and were manually reviewed from the hospital database. The severity of the postoperative somatic general complication was classified according to the Clavien-Dindo classification (CDC) (29). The CDC grades were not applied to psychiatric complications. Psychiatric complications were defined as new or worsened severe psychiatric morbidity occurring during the postoperative follow-up requiring medical evaluation, pharmacological treatment, hospitalization, or other therapeutic intervention. Assessment was based on documented psychiatric diagnoses, patient-reported mental health symptoms, and initiation or modification of psychiatric medication. The severity was classified according to the degree of functional limitation of the mental illness (30).

The predictor variables were age, gender (female/male), body mass index (BMI), long-term disease(s) requiring regular intervention or medication (cardiovascular disease, severe psychiatric morbidity, lung disease, diabetes I/II, autoimmune disease, endocrinologic disease, moderate or severe sleep apnoea, or other), smoking, and alcohol and/or drug abuse. Alcohol and/or drug abuse history was determined according to the Finnish Current Care Guidelines (31). Surgery-related variables were surgical procedures classified as BSSO, Le Fort I, or bimaxillary-osteotomy and administration of dexamethasone grouped as 10 mg or &gt;10 mg.

Ethical considerations

The study protocol was approved by the Internal Review Board of the Head and Neck Centre, Helsinki University Hospital, Finland (HUS/355/2025). The principles outlined in the Declaration of Helsinki were followed.

Statistical analysis

Analysis was conducted using SPSS software (IBM Corporation, 28.0.0.0). Pearson's chi-squared test was used to assess differences between the patients grouped by their categorical variables. For the applicable variables, means, minimums, maximums, and medians were computed. To investigate the relationship between the variables, logistic regression analysis was employed. The variance inflation factor (VIF) analysis revealed no multicollinearity among the predictor variables. Consequently, all predictor variables were included in a multivariable model. For analysis, a significance level of .05 was chosen.

## Results

The study population included 452 patients, and after inclusion and exclusion criteria, 429 patients (42% men, 58% women) remained in the final analyses. The most common surgery type was BSSO exclusively, and patients' perioperative age ranged from 19 to 61 (mean 33.0, median 30) years (Table 1). Most patients (70%) did not have pre- or perioperative long-term disease.

Table 1

A total of 16 patients (3.7%) had a non-surgical site complication within 90 days after surgery, and a total of 19 complications were recognized (Table 1, Table 2).

Table 2

The most common complication was severe psychiatric morbidity (37%), followed by respiratory (21%), cardiovascular (16%), and neurological (11%) complications. Based on the CDC grade (29), the majority of patients had complications classified as grade I or II (60%). Grade I complications included one patient with gastroenteritis, two patients with peroneus paresis, and one patient with atelectasis leading to a decrease in saturation and requiring therapeutic intervention. Grade II included one patient with deep venous thrombosis and two patients with pulmonary embolism: One with lung infarction and one without. Three were classified as potentially life-threatening complications (CDC grades IIIa-Iva) needing surgical, endoscopic, or radiological intervention or intensive care/intensive care unit (IC/ICU) management (30%), and these potentially life-threatening complications occurred in .7% of all patients. Grade IIIa included one patient with urinary retention requiring the insertion of an indwelling catheter. Grade Iva included one patient with high blood pressure and bleeding who required multiple pharmacological interventions and plasma transfusion with ICU management, and one patient with aspiration pneumonia with high C-reactive protein (CRP) concentration in blood and fever over 38.5°C, deep vein thrombosis, and pulmonary embolism requiring ICU management. Patients with cardiovascular and respiratory complications and thromboembolism were older than patients in other complication subgroups.

Univariate and multivariable logistic regression analyses are presented in Table 3.

Table 3

In univariate logistic regression analyses, patients with preoperative long-term disease (OR 5.435; 95% CI 1.849-15.979; p=.002) and preoperative alcohol and/or drug abuse (OR 7.712; 95% CI 1.939-30.667; p=.004) were more likely to have a postoperative complication. These variables predicted the outcome independently in the multivariable logistic regression model. Patients with long-term diseases had 4.8 times higher odds (aOR 4.820; 95% CI 1.541-15.071, p=.007) of having a postoperative general complication, and alcohol and/or drug abuse was also associated with the outcome (p=.031). Surgery type or other explanatory variables were not statistically significant.

Associations between long-term diseases and the outcomes are presented in Table 4. The majority of patients with postoperative general complications had a long-term disease before surgery (p=.001). Severe psychiatric morbidity was significantly associated with the outcome (p&lt;.001).

Table 4

## Discussion

The aim of this retrospective study was to evaluate the occurrence of non-surgical site complications in patients receiving OS. We hypothesized that the spectrum of complications outside the surgical site is broad and that we would identify new general complications warranting medical care or intervention. The results confirmed our hypothesis. Severe psychiatric complications are rare (1.6% of all patients); together, however, they comprise a high proportion of all reported general complications (37%, Table 2). Of the predictor variables, long-term diseases predicted postoperative complications independently, and presurgical psychiatric morbidity was associated with the outcome.

The rate for non-surgical site complications was 3.7% (Table 1), which is lower than Riekert et al. (25) reported in their study (6.8%) and slightly higher than reported by Ferri et al. (32) (1.5%). In the present study, the types and severities of somatic complications varied, and the number of specific complication subtypes remained low. Notable was, however, the occurrence of severe psychiatric morbidity. Postoperative mental health impairment leading to severe mental health disorder was the most frequent complication type, affecting 1.6% of all patients (Table 2).

Previously, we highlighted the role of psychiatric disorders and the exacerbation of psychiatric diseases in patients undergoing OS (20). Severe psychiatric complications have not earlier been reported alongside somatic complications. Patients' quality of life does not necessarily improve after OS (33), and depression negatively affects oral health-related quality of life (34). Here, postoperative severe psychiatric complications were rare; however, these complications included suicidal ideation, severe depression, severe anxiety, self-harm, and drug overdose at the early stage of recovery (Table 2).

Based on our results, the occurrence and relevance of psychiatric complications are greater than previously understood. In the literature, OS patients have often had preceding psychiatric morbidity (20 , 35 , 36). In our study, long-term disease predicted postoperative general complications, and of these long-term diseases, preceding severe psychiatric morbidity was especially associated with the outcome. It has also been noted that early-stage postoperative morbidity, such as lack of sleep, nausea, and vomiting, can impair postoperative psychiatric recovery (37). This emphasizes that more attention should be paid to examining patients' mental well-being at all phases of OS treatment.

The overall complication rate for somatic complications was 2.8%, and the rate for potentially life-threatening complications remained low (.7%, Table 2). No deaths were reported in our study. In all, 1.6% of OS patients suffered from cardiovascular, thromboembolic, or respiratory complications (Table 2), corresponding to the figure in Van De Perre et al. (18) (1.5%). In the literature, potentially life-threatening complications have been described. Panula et al. (8) reported an occurrence of bleeding of 0.02%, and occurrence of severe infections in OS has varied from 0.4% to 3.3% (12 , 18 , 38). In the present study, two patients required ICU treatment: One patient with high blood pressure and bleeding requiring plasma transfusion (.2% of all patients) and the other with aspiration pneumonia and pulmonary embolism (.2% of all patients).

In earlier reports, the extent of surgery and the duration of the procedure affect the risk of complications. Bimaxillary surgery has been noted to be associated with a higher risk for general complications than single-jaw surgery (39 , 40). In this study, we found no association between the type of surgery and the outcome (Table 3). As reported also previously in OS, smoking has been cited as a significant risk factor for surgical site infections (14 , 41), however, we found no association between smoking and non-surgical site complications. However, smoking is known to increase local and general complications, and it is therefore justified to encourage patients to quit smoking early before OS, already during orthodontic treatment.

Even though age was not found to be associated with the complication outcome, we did find differences in the median and mean ages when comparing subgroups of general complications. Age may be related to the complication type, considering that cardiovascular, respiratory, and thromboembolic complications were more common in older patients (Table 2). These groups were, however, too small for statistical analyses and warrant further research before conclusions can be drawn.

In this study, drug and alcohol abuse were independent predictors of postoperative problems (Table 3). They have been linked to postoperative infections in the surgical site and have been shown to increase the risk of impaired healing in maxillofacial surgery (42 , 43). Preoperative high alcohol consumption has been linked to increased risk for pulmonary complications, general infections, length of hospital stay, and ICU admission (44). Considering these findings, a comprehensive review of preoperative alcohol use is necessary when developing an OS treatment plan, and alcohol use must be addressed.

A high dose of dexamethasone has been associated with more major complications in oral cancer patients with microvascular reconstruction (45), and administration of dexamethasone has also been associated with higher short-term mortality in cancer patients receiving reconstructive head and neck surgery (46). Dexamethasone also seems to negatively affect the union in mandibular fractures (47) and wound healing in patients undergoing surgery for zygomatic fractures (48). In this study, most patients with complications received a higher dose of perioperative dexamethasone (Table 4), but the difference remained statistically insignificant. However, according to results from previous studies, a high dose of dexamethasone should be used with caution (20).

Some limitations of this study should be addressed. This is a single-centre study as well as a retrospective study. The data were collected over a long period, no standardized protocols were in place for all surgery-related procedures and medications. Some information might be missing due to practitioners' documentation in the patient data system. Complications may be underrated, especially regarding psychiatric morbidity, as we do not have a systematic evaluation system for patients' mental health status prior to surgery. It also should be noted that the number of complications was low relative to the number of covariates included in the multivariable model, which may have reduced statistical power and increased uncertainty in the adjusted estimates. Future studies with larger cohorts or penalized regression methods could provide more stable outcome estimates.

## Conclusions

The results of this cohort study suggest that non-surgical site complications are rare in patients undergoing OS (3.7%), however, severe complications can occur. We suggest that thorough evaluation of both somatic and psychiatric diseases should be carried out before the surgery. Emphasis should be placed on patients' psychiatric status both pre- and postoperatively, as our findings indicate that psychiatric morbidity is the most frequently found severe complication in this patient group.

## Figures and Tables

**Table 1:** Table Descriptive statistics of 429 patients receiving orthognathic surgery.

	No. of patients		% of 429
AllAge (years) Range Mean±SD Median	429 19-6133.0±10.0130		
Gender			
Male	181		42.2
Female	248		57.8
Any long-term disease(s)			
Yes	130		30.3
No	299		69.7
Cardiovascular disease	36		8.4
Severe psychiatric morbidity	22		5.1
Lung disease	44		10.3
Diabetes	5		1.2
Autoimmune disease	15		3.5
Endocrinological disease	16		3.7
Moderate or severe sleep apnoea	17		4.0
Other*	8		1.9
Smoking			
Yes	68		15.9
No	361		84.1
Alcohol and/or drug abuse			
Yes	15		3.5
No	414		96.5
Body mass index			
Range		16.3-36.4	
Mean±SD		24.3±3.79	
Median		23.7	
Surgery type			
Bilateral sagittal split osteotomy	182		42.4
Le Fort I osteotomy	102		23.8
Bimaxillary osteotomy	145		33.8
Dexamethasone			
<=10 mg	252		58.7
>10 mg	177		41.3
Non-surgical site complication			
Yes	16		3.7
No	413		96.3

SD: Standard deviation. *Ehler-Danlos syndrome, human immunodeficiency virus (HIV) infection, epilepsy.

**Table 2:** Table Descriptive statistics of 16 patients with 19 non-surgical site complications from among the 429 orthognathic surgery patients.

Complication type	n	% of 429 patients	% of all complications (n=19)	Median age	Mean age	Detailed description
Cardiovascular	1	.2	5	58.7	58.7	High blood pressure and bleedingrequiring plasma transfusion
Respiratory	2	.5	11	50.9	50.9	Pulmonary infarction due to pulmonary embolism, atelectasis with decrease in saturation requiring therapeutic intervention
Thromboembolism	4	.9	21	42.6	40.8	Deep venous thrombosis andpulmonary embolism
Psychiatric	7	1.6	37	31	31	Suicidal ideation, suicidal self-injury, suicidal drug overdose, severe panic attack, severe anxiety, severe depression
Gastrointestinal	1	.2	5	31	31	Gastroenteritis
Renal	1	.2	5	28	28	Urinary retention requiring anindwelling catheter
Neurological	2	.5	11	25	25	Peroneus paresis
General infection	1	.2	5	23	23	Pneumonia with high C-reactiveprotein concentration in blood andfever over 38.5°C
Clavien-Dindo classification grade (excluding psychiatric complications) [29]	n	% of 429 patients	% of all patients with somatic complications	Median age	Mean age	Detailed description
I	4	.9	40	28	31.8	Any deviation from the normal postoperative course without need for pharmacological treatment or surgical, endoscopic, or radiological intervention
II	3	.7	30	42.5	41.7	Requiring pharmacological treatment with drugs other than such allowed for grade I complications. Blood transfusions andtotal parenteral nutrition are also included
IIIa	1	.2	10	27.7	27.7	Requiring surgical, endoscopic, or radiological intervention, interventionnot under general anaesthesia
IIIb	0	0	0	0	0	Requiring surgical, endoscopic or radiological intervention, interventionunder general anaesthesia
IVa	2	.5	20	40.6	40.6	Life-threatening complication (including CNS complications)* requiring IC/ICU management, single-organ dysfunction
IVb	0	0	0	0	0	Life-threatening complication (including CNS complications)* requiring IC/ICU management, multiorgan dysfunction
V	0	0	0	0	0	Death of a patient

CNS: Central nervous system. IC/ICU: Intensive care/intensive care unit.

**Table 3:** Table Logistic regression model explaining complication occurrence with predictor variables.

Univariate logistic regression analyses						Multivariate logistic regression analyses				
Variable	Coefficient	SE	OR	95% CI	p	Coefficient	SE	aOR	95% CI	p
Age (years)	.021	.024	1.021	.975-1.070	.378	.007	.027	1.007	.955-1.062	.791
Alcohol and/or drug abuse	2.043	.704	7.712	1.939-30.667	.004	1.710	.792	5.529	1.170-26.124	.031
Gender	.066	.514	1.068	.390-2.924	.898	-.280	.566	.756	.249-2.290	.621
Smoking	.926	.556	2.525	.848-7.516	.096	.251	.636	1.285	.370-4.466	.693
Surgery type BIMAX (ref. single jaw surgery)	.438	.515	1.550	.565-4.250	.395	.636	.555	1.889	.631-5.607	.252
Long-term disease	1.693	.550	5.435	1.849-15.979	.002	1.573	.582	4.820	1.541-15.071	.007
Body mass index	.086	.062	1.090	.965-1.231	.166	.082	.066	1.086	.954-1.236	.213
Dexamethasone >10mg (ref. <=10mg)	.898	.526	2.455	.876-6.884	.088	.856	.559	2.353	.787-7.033	.126

CI: Condifence interval. OR: Odds ratio. SE: Standard error. aOR: Adjusted odds ratio.

**Table 4:** Table Associations between long-term diseases, dexamethasone and non-surgical site complications in 429 orthognathic surgery patients.

	Patients with complication		Patients without complication		p	Effect sizeif significant
	n	%	n	%		
All	16	3.7	413	96.3		
Any long-term disease					.001	.165
Yes	11	8.5	119	91.5		
No	5	1.7	294	98.3		
Cardiovascular disease	1	2.8	35	97.2	1.000	
Severe psychiatric morbidity	2	9.1	20	90.9	<.001	.400
Lung disease	1	2.3	43	97.7	.674	
Diabetes	1	20.0	4	80.0	.174	
Autoimmune disease	1	6.7	14	93.3	.440	
Endocrinological disease	0	0	16	100	1.000	
Moderate or severe sleep apnoea	2	11.8	15	88.2	.128	
Other*	0	0	8	100	1.000	
Dexamethasone					.079	
<=10 mg	6	2.4	246	97.6		
>10 mg	10	5.6	167	94.4		

SD: Standard deviation. ns: Non-significant. *Ehler-Danlos syndrome, human immunodeficiency virus (HIV) infection, epilepsy.

## Data Availability

Declared none.
